# Abundance and Antimicrobial Resistance of Three Bacterial Species along a Complete Wastewater Pathway

**DOI:** 10.3390/microorganisms7090312

**Published:** 2019-09-03

**Authors:** Ilse Verburg, Silvia García-Cobos, Lucia Hernández Leal, Karola Waar, Alex W. Friedrich, Heike Schmitt

**Affiliations:** 1 Wetsus, European Centre of Excellence for Sustainable Water Technology, 8900 CC Leeuwarden, The Netherlands (I.V.) (L.H.L.); 2 Department of Medical Microbiology and Infection Prevention, University of Groningen, University Medical Center Groningen, 9713 GZ Groningen, The Netherlands (S.G.-C.) (A.W.F.); 3 Izore, Centrum Infectieziekten Friesland, 8900 JA Leeuwarden, The Netherlands; 4 Institute for Risk Assessment Sciences, Utrecht University, 3508 TD Utrecht, The Netherlands; 5 Centre for Infectious Disease Control, National Institute for Public Health and the Environment (RIVM), 3721 MA Bilthoven, The Netherlands

**Keywords:** antimicrobial resistance, wastewater pathway, *Escherichia coli*, *Klebsiella* spp., *Aeromonas* spp., bacterial abundance, antimicrobial consumption, clinical wastewater, wastewater treatment plant, antimicrobial residues

## Abstract

After consumption, antibiotic residues and exposed bacteria end up via the feces in wastewater, and therefore wastewater is believed to play an important role in the spread of antimicrobial resistance (AMR). We investigated the abundance and AMR profiles of three different species over a complete wastewater pathway during a one-year sampling campaign, as well as including antimicrobial consumption and antimicrobial concentrations analysis. A total of 2886 isolates (997 *Escherichia coli*, 863 *Klebsiella* spp., and 1026 *Aeromonas* spp.) were cultured from the 211 samples collected. The bacterial AMR profiles mirrored the antimicrobial consumption in the respective locations, which were highest in the hospital. However, the contribution of hospital wastewater to AMR found in the wastewater treatment plant (WWTP) was below 10% for all antimicrobials tested. We found high concentrations (7–8 logs CFU/L) of the three bacterial species in all wastewaters, and they survived the wastewater treatment (effluent concentrations were around 5 log CFU/L), showing an increase of *E. coli* in the receiving river after the WWTP discharge. Although the WWTP had no effect on the proportion of AMR, bacterial species and antimicrobial residues were still measured in the effluent, showing the role of wastewater contamination in the environmental surface water.

## 1. Introduction

Antimicrobials are essential to treat bacterial infections. The emergence and spread of antimicrobial resistance (AMR) has been globally recognised as a serious threat to public health [1]. Additionally, antimicrobial consumption can lead to alterations in the human microbiota and can select for antimicrobial resistant bacteria (AMRB). These AMRB and partially metabolized antimicrobials are excreted into the wastewater with urine and feces and may end up in surface water via the sewage system.

Clinical environments, such as hospitals and nursing homes, are hotspots for AMRB [2], however >80% of the total antimicrobial consumption in the human sector is prescribed in the community in Europe [3,4]. Although recent studies demonstrated that hospital wastewater contains higher levels of AMRB, antimicrobial residues and antimicrobial resistance genes (ARGs) [5,6,7], the impact of hospital wastewater on antimicrobial resistance found in the receiving wastewater treatment plant (WWTP) varies per study [5,6]. The question therefore arises whether on-site clinical wastewater treatment is effective in reducing the release of AMR. Also, the contribution of healthcare institutions and the general community as sources of AMRB might depend on the bacterial species investigated and its prevalence in both the general community and healthcare institutions.

WWTP collect domestic wastewater, clinical wastewater, and wastewater from industry. The main goal of WWTPs is to reduce nitrogen, phosphorus, total suspended solids, and the biological oxygen demand, but WWTPs are not designed to remove bacteria and micropollutants such as antimicrobial residues and heavy metals. In the WWTP, many different bacteria are mixed together with pollutants, creating circumstances that might favour horizontal gene transfer and selection of AMRB [8,9]. Thus, WWTPs may contribute to the spread of AMRB into the receiving surface water [10]. 

Contradictory results have been found on the results of WWTP treatment for resistance. The proportion of AMR has been found to increase [11,12,13] as well as decrease [14,15,16] in the effluent. However, in a recent large study, the same percentage of AMR *Escherichia coli* was observed in WWTP influent and effluent [17]. Again, the role of species identity for AMR selection through the WWTP is yet unknown. 

In this study, three different bacterial species were investigated. *E. coli* is a typical gut bacteria [18] which has been the foremost indicator for monitoring faecal contamination in water [19]. *Klebsiella* spp., is a common hospital-related pathogen, which has been scarcely investigated in (waste)water. Multidrug resistant *E. coli* and *Klebsiella* spp. are both important human pathogens. In addition, several ARGs were first discovered in *K. pneumoniae*, and probably this species plays a key role in the dissemination of AMR [20]. *Aeromonas* spp. are abundant in different aquatic environments, they have also been described as opportunistic pathogens for humans and (aquatic) animals [21], and they are considered as important vectors for ARGs into the environment [22,23]. 

The aim of this study was to investigate the AMRB contribution of clinical and non-clinical wastewater sources to the wastewater chain and their dependence on the antibiotic usage, the fate of AMRB in the treatment plant, and the impact of WWTP discharges in terms of AMR on the receiving surface water. For this purpose, the abundance and antimicrobial susceptibility of three bacterial species, *E. coli*, *Klebsiella* spp., and *Aeromonas* spp., collected from several (waste)water locations: community, hospital, nursing home, influent and effluent of WWTP, and two surfaces waters, were studied during a one-year sampling campaign. In addition, results were analysed in relation to the antimicrobial consumption and antimicrobial concentrations in the water samples.

## 2. Materials and Methods 

### 2.1. Sampling Locations and Sample Collection

Wastewater samples were taken in the city of Sneek, The Netherlands (33,855 inhabitants) including the following locations: wastewater from a nursing home (220 beds), a hospital (300 beds), a community wastewater collection point (80 households), and the influent and effluent of the corresponding municipal WWTP. The wastewater samples from the community, hospital, and nursing home were taken from the receiving wells of which neither receive other wastewater nor rainwater. Municipal wastewater (including industrial wastewater and rain) is treated centrally at a conventional WWTP (aerobic treatment, 73,000 p.e.), and the effluent is discharged into the Geeuw canal. Twenty-four-hour samples were collected flow proportionally (WWTP) or time proportionally (for community, hospital, and nursing home). When a technical failure occurred, a grab sample was taken, which happened once for influent, community, and hospital and three times for effluent. Grab samples from the wastewaters and effluent were excluded from the bacterial concentration calculations and the measurements of antimicrobial residues and chemical concentrations, but these samples were still used to obtain bacterial isolates for antimicrobial susceptibility testing. Surface water samples were collected from the receiving surface water of the Geeuw canal at two locations, 330 m south-west (N 53°02′15.10″, E 5°63′72.76″) and 388 m north-east (N 53°02′72.15″, E 5°64′28.97″) from the WWTP discharge point (N 53°02′38.85″, E 5°64′03.20″). The flow direction of the receiving surface water is artificially managed and can change per hour. In order to state if the sample location was up- or downstream from the WWTP discharge at the time of sampling, information was obtained from the waterboards. A sampling point was distinct up- or downstream if the direction of the waterflow had not changed in the 24 h before sampling. Out of 52 samples collected from the receiving surface water, 26 could be defined as up- or downstream. In addition, surface water samples were collected from a non-receiving surface water located in a nature reserve “de Deele” (N 53°01′91.00″, E 5°92′00.72″), which was expected to be barely effected by anthropogenic activities, and was thus considered as a control surface water [24]. Surface water samples were collected as grab samples at a one-meter distance from the waterside and ~20 cm depth. The samples were collected in 2017, every four weeks, two days in a row in 2-L high density polyethylene (HDPE) bottles (VWR, Amsterdam, The Netherlands), and transported cooled to the laboratory where they were processed the same day.

### 2.2. Bacterial Identification and Enumeration 

Each sample was filtered in a gradient of volumes [25] using the EZ-Fit ^TM^ filtration system with 0.45 μm pore size membrane filters (EX-Pak filters, Millipore, Burlington, MA, USA). Membranes were transferred onto: (a) Simmons citrate agar (Oxoid) to culture *Klebsiella* spp., followed by incubation for 40–48 h at 37 °C; (b) *Aeromonas* medium base (RYAN) agar (Oxoid) to culture of *Aeromonas* spp., followed by incubation for 16–24 h at 37 °C. After enumerating the colony forming units (CFU), five to eight presumptive colonies of *Klebsiella* spp. and *Aeromonas* spp. were isolated from each sample and species identification was confirmed using MALDI-TOF MS (BioMérieux Vitek MS). Up to five of the confirmed isolates per sample were stored for further analysis. A total of 74–87.7% of the tested isolates were confirmed as *Aeromonas* spp., and 70.2–88.3% as *Klebsiella* spp. in the wastewaters, the WWTP, and receiving surface waters. However, the percentage of confirmed *Klebsiella* spp. in the control surface water was rather low (10.75%), and only eight isolates (6.2%) of *Klebsiella* spp. were obtained from this location. The *Klebsiella* spp. identified were either *K. pneumoniae* or *K. oxytoca*, the total amount of *Klebsiella* spp. was used for the bacterial enumeration. For *Aeromonas* spp., bacteria could not be distinguished at species level. Tryptone Bile X-Glucuronide medium (TBX) (Oxoid) was used to culture *E. coli*, and after 16–24 h incubation at 37 °C all blue/green colonies were counted. At random, 159 (16%) isolates collected from wastewater-samples, WWTP, and receiving surface waters (16–33 isolates per sample) were tested by MALDI-TOF, and 96.7–100% was identified as *E. coli*. However, in the control surface water, the percentage of confirmed *E. coli* isolates was lower (76.3%). Plates containing up to 100 CFU (*Klebsiella* spp. and *Aeromonas* spp.) or 150 CFU (*E. coli*) were included for the enumeration. CFU counts were adjusted to the percentages of the confirmed isolates to determine the bacterial concentrations [25]. Screening and extra CFU counts for extended-spectrum beta-lactamase (ESBL)-producing *E. coli* (ESBL-EC) were performed using a chromogenic media (BioMérieux) for three months (September, October, and November of 2017). The suspected ESBL-EC colonies were not further confirmed. 

### 2.3. Antimicrobial Susceptibility Testing 

Five isolates of each bacterial species were collected per sample to perform antimicrobial susceptibility testing (AST), except for the WWTP influent and the surface waters in which it was not always possible to obtain five isolates of each species per sample. The bacterial isolates were subjected to AST using the disk diffusion method and the results were interpreted following the European Committee on Antimicrobial Susceptibility Testing (EUCAST) criteria [26]. The same breakpoint were applied to *Aeromonas* spp. as for *Enterobacteriaceae* [27]. Multi drug resistance (MDR) was defined according to Magiorakos et al. [28], with resistance against at least one agent in three or more antimicrobial categories. *E. coli* and *Klebsiella* spp. isolates were tested using twelve antimicrobials from six classes: (1) Penicillins: ampicillin (AMP) and amoxicillin + clavulanic acid (co-amoxiclav, AMC); (2) cephalosporins: cefuroxime (CXM), ceftazidime (CAZ), and cefotaxime (CTX); (3) folate pathway inhibitors: trimethoprim (W) and co-trimoxazole (SXT); (4) fluoroquinolones: ciprofloxacin (CIP); (5) carbapenems: meropenem (MEM); and (6) aminoglycosides: gentamicin (CN) and tobramycin (TOB). Isolates that showed resistance against ceftazidime and/or cefotaxime, were further investigated for ESBL phenotypes using the Vitek 2 system with gram negative susceptibility card AST-N344 (BioMérieux) and confirmation by double disk diffusion. *Aeromonas* spp. susceptibility was tested for six antimicrobials (all from different classes): ceftazidime (CAZ), co-trimoxazole (SXT), ciprofloxacin (CIP), meropenem (MEM), gentamicin (CN), and piperacillin (PRL). In addition, information on AST of *K*. *pneumoniae* and *E. coli* clinical isolates collected from the urine of patients from the investigated hospital (*n* = 544 for *E. coli* and *n* = 131 for *Klebsiella* spp.), nursing home (*n* = 113 for *E. coli* and *n* = 22 for *Klebsiella* spp.), and from general practitioners (GPs) in Sneek (*n* = 403 for *E. coli* and *n* = 66 for *Klebsiella* spp.), was gathered for comparison purposes. Resistance data were collected for each species of one isolate per patient per year.

### 2.4. Antimicrobial Consumption

Data on antimicrobial consumption in the hospital and nursing home in 2017, from which the wastewater was sampled, was provided by the hospital pharmacist. The amount of used antimicrobials was calculated as defined daily doses (DDD) using the Anatomical Therapeutic Chemical (ATC) classification system for medicines from the WHO, version 2018 [29]. The annual number of bed-days and admissions in the hospital were obtained from the societal annual report from the hospital [30], and corrected as reported by Liem et al. [31]. The antimicrobial use at community level was obtained from the annual Nethmap report [32], using the values of 2016. The antimicrobial use in the hospital, nursing home, and community is presented in DDD/1000 patient-days (hospital), resident-days (nursing-home), or inhabitant-days (community).

### 2.5. Statistical Analyses

We applied the two-sample *t*-test assuming unequal variances to determine the differences in CFU-counts between two locations, using Microsoft Excel (Office 365). The difference in bacterial concentrations between two different locations was considered significant when *p* > 0.05 and *t*-value > *t*-critical. For the differences in AMR found between two locations, univariate analyses were performed using the Fisher’s exact or Chi-square (including Yates correction) methods for categorical variables using Minitab (version 17).

### 2.6. Contribution of Hospital Wastewater to AMRB in Influent

The contribution of hospital wastewater to AMRB in influent was calculated using the following equation:

(H_conc_ × H_total-volume_ × H_R_):(I_conc_ × I_total-volume_ × I_R_), (1)

in which H_conc_ is the concentration of bacteria (CFU/L) in hospital and I_conc_ in influent, H_total-volume_ is the total volume of wastewater (m^3^/year) produced by hospital and I_total-volume_ processed by the WWTP, and H_R_ is the percentage of resistant bacteria against a certain antimicrobial in hospital and I_R_ in influent. Antimicrobials with only one resistant isolate were not included in the calculation.

### 2.7. Antimicrobial Residues and Chemical Concentrations

The antimicrobial residues and chemicals were measured by liquid chromatography coupled to a triple–quadruple mass spectrometer (LC/MS–MS, Agilent Technologies 1200 and 6410 series). A sample of 2 mL was mixed for 30 min (1750 rpm) with 0.2 mL methanol and 0.1 mL modifier (12.8% formic acid, 0.24 M NH_3_, and 0.02 M oxalic acid). After 10 min of centrifugation (3773 rcf), 900 µL of the liquid phase was mixed with 50 µL internal standard (containing trimethoprim-d9, triclosan, fenoprofen, atenolol, ciprofloxacin-d8, sulfadoxine-d3, and diaveridine) and either 50 µL of milliQ water or 50 µL of additional standard (containing 6.8 µg of the compound to be measured). Antimicrobials to be measured were selected based on the antimicrobial consumption in The Netherlands in 2016 [32]. The selected antibiotics comprised: (1) macrolides: azithromycin, clarithromycin, erythromycin, tilmicosin, and tilosin; (2) (synthetic) fluoroquinolones: ciprofloxacin, ofloxacin, and flumequine; (3) lincosamides: lincomycin and clindamycin; (4) folate pathway inhibitors: trimethoprim and sulphamethoxazole; (5) penicillins: amoxicillin, ampicillin, penicillin G and V; (6) cephalosporins: cefotaxime; (7) tetracyclines: tetracycline, doxycycline, and oxytetracycline; (8) sulfonamides: sulphadoxin, sulphachloropyridazine, sulphapyridine, and sulphamethazine. The disinfectants chlorhexidine and triclosan were also measured. From the quaternary ammonium compounds, benzalkonium chloride (BAC)12 and BAC14, which are often applied in disinfectant products, were measured. In addition, three potential human markers were measured: gabapentin, sucralose, and acesulfame. The measured values were multiplied by their recovery rates, which should be in the range of 60–140%. If recovery rates were not in the range of 60–140%, the measured values were excluded. The heavy metals copper, cadmium and zinc were measured by ICP-OES using the Perkin Elmer Optima 5300 DV. When a compound was not measured above the limit of detection (LOD), the value of LOD/√2 was used.

## 3. Results and Discussion

In total, 211 samples were collected during the sampling campaign in 2017, from which 2886 isolates (997 *E. coli*, 863 *Klebsiella* spp., and 1026 *Aeromonas* spp.) were obtained. The total number of samples obtained per location, and the total number of isolates per bacterial species obtained in each location is shown in Table S1.

### 3.1. Antibiotic Consumption and Measured Concentrations Were Highest in Hospital 

**Figure 1:**
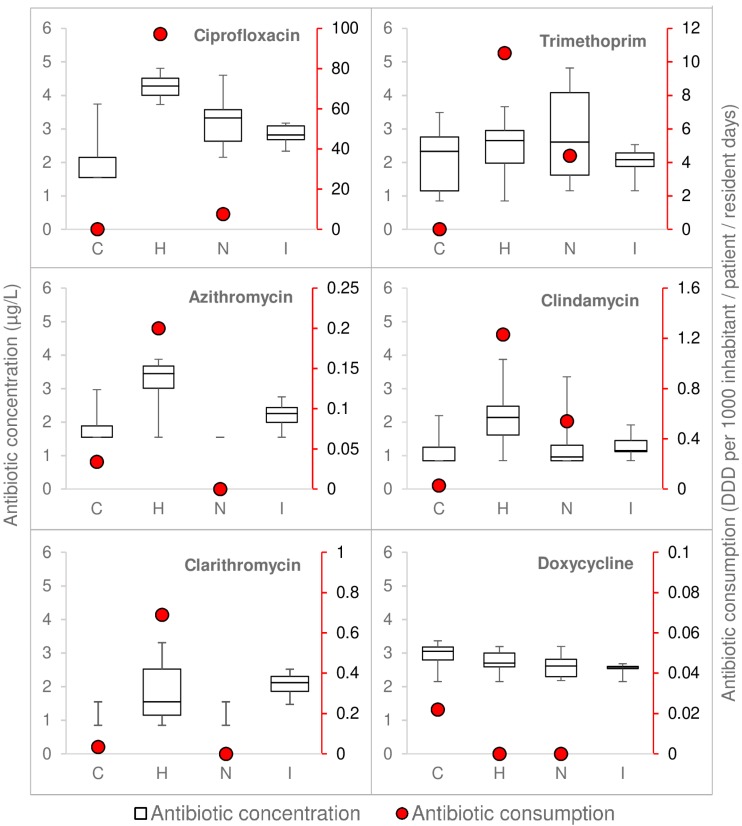
Antimicrobial consumption versus the measured concentrations. The red dots display the antimicrobial consumption, and the boxplots show the antimicrobial concentration measured in the wastewater. C = community, H = hospital, N = nursing home, I = influent. The antimicrobial concentrations measured in community (azithromycin, clindamycin, and clarithromycin) and nursing home wastewater (azithromycin and clarithromycin) are not shown as these locations contained less than five samples with decent measurements.

In general, both antimicrobial consumption and measured concentrations of antimicrobial residues in wastewater were higher in hospital wastewater (Figure 1, Table S2) than wastewater from the nursing home and the community. It should be noted that the community sampled in this study (80 households) was only a small part of the whole community, and therefore it is discussable whether this was representative. The highest trimethoprim consumption was observed in the hospital, however measured trimethoprim concentrations were occasionally higher in nursing home wastewater than in hospital wastewater.

Although consumption of penicillins was relatively high in both hospital and nursing home (not shown), these antimicrobials could not be measured in any of the wastewater samples, because of their chemical instability [33]. Tetracycline was not used in the hospital, but still it was found at low concentrations in hospital wastewater (six samples) (Table S2). These tetracycline residues could originate from outpatients visiting the hospital, as tetracyclines are the second most consumed antimicrobial group in the community in The Netherlands [4]. 

### 3.2. Concentrations of Resistant Bacteria Mirrored Antimicrobial Consumption 

**Figure 2:**
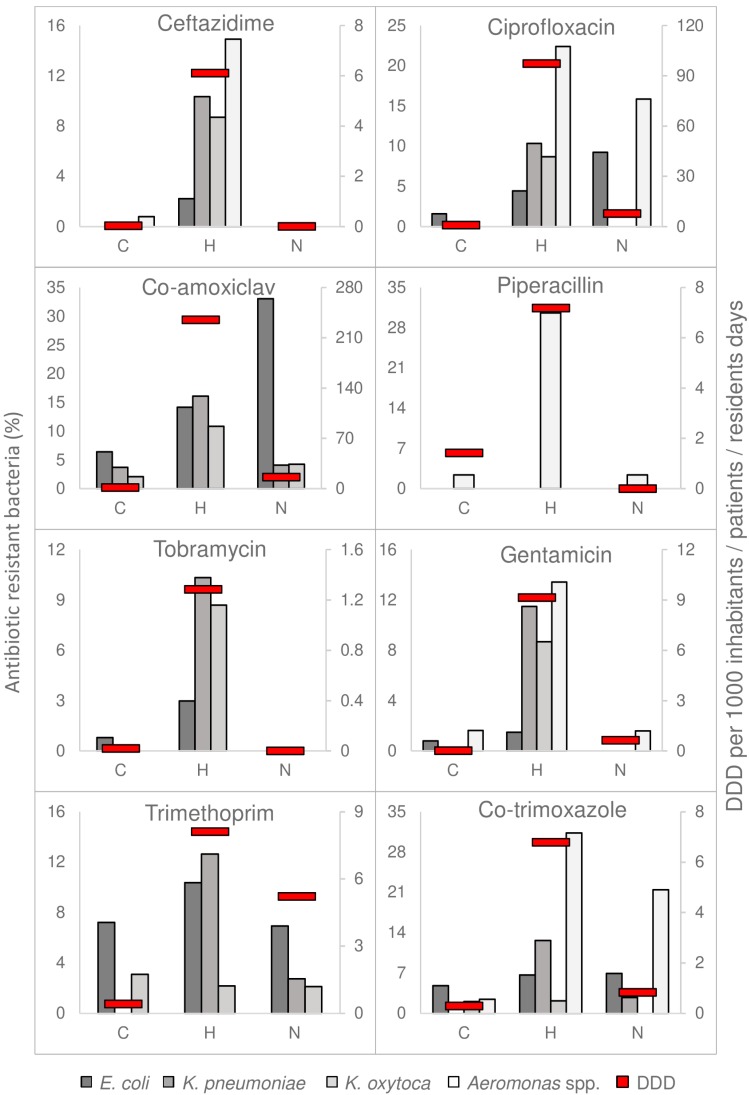
Antimicrobial consumption versus resistance. The antimicrobial consumption in daily defined dose (DDD) per 1000 inhabitant/patient/resident-days is shown as red bars. The grey bar plots represent the percentage of antimicrobial resistant bacteria measured in the wastewater. C = community, H = hospital, and N = nursing home (N). Meropenem (not shown) was only used in the hospital, although at low amounts (0.47 DDD/1000 patient-days). All bacterial isolates were susceptible to meropenem.

The proportion of AMRB found in wastewater samples generally mirrored the antimicrobial consumption in the corresponding location (Figure 2). 

Proportions of resistant *K. pneumoniae* (for resistance to ceftazidime, ciprofloxacin, co-amoxiclav, tobramycin, gentamycin, trimethoprim, and co-trimoxazole), *K. oxytoca* (ceftazidime, ciprofloxacin, co-amoxiclav, tobramycin, gentamycin) were higher in hospital wastewater, in line with the consumption of these antimicrobials. With the exception of trimethoprim and co-trimoxazole, the percentage of resistant *K. oxytoca* isolates (including MDR) was significantly higher in hospital wastewater than in communal wastewater (*p* < 0.05). Differences in the percentages of resistant *K. pneumoniae* between hospital and communal wastewater were not significant, this may be due to the low number of isolates retrieved from communal wastewater (*n* = 27).

The percentage of resistant *Aeromonas* spp. (including MDR) was significantly higher in the hospital than in communal wastewater (*p* < 0.001). In addition, co-trimoxazole and ciprofloxacin resistance in *Aeromonas* spp. from nursing home wastewater was significantly higher than from communal wastewater (*p* < 0.001). 

In contrast to *Klebsiella* and *Aeromonas* spp., the differences observed in resistance percentages of *E. coli* between clinical and non-clinical wastewater were smaller. Still, resistance against ampicillin and co-amoxiclav was significantly higher in hospital and nursing home wastewater than in communal wastewater (*p* < 0.05 and *p* < 0.001, respectively). In addition, ciprofloxacin resistant *E. coli* in nursing home wastewater were significantly higher than in communal wastewater (*p* < 0.01). The percentage of MDR-*E. coli* was low in all the samples (3% and lower). 

In both hospital and nursing home, AMR was generally higher in clinical isolates obtained from patients than in wastewater isolates, especially for *E. coli* (Figures S1 and S2). AMR in *E. coli* and *Klebsiella* spp. isolates from GPs in Sneek was also higher than in wastewater isolates, and more similar to AMR observed in clinical isolates. AMR observed in wastewater therefore reflected AMR in the general population rather than in clinical patients. Still, resistance in wastewater isolates and in clinical isolates showed the same trends between hospital, nursing home, and the general community. 

The smaller difference in resistance percentages of *E. coli* in clinical and non-clinical wastewater, and the bigger difference in resistance percentage of *E. coli* between clinical isolates and wastewater isolates, suggest that clinically-related *E. coli* abundance in wastewater would be diluted by *E. coli* from healthy staff and visitors and from patients who are not using antimicrobials. *E. coli* is part of the normal gut microbiota [18], in contrast to *Klebsiella* and *Aeromonas* spp. which are found in only a part of the human population and linked to diseases associated with gut microbiome dysbiosis [34,35,36,37].

### 3.3. ESBL in Wastewater 

All the MDR-*Klebsiella* spp. isolates found in hospital wastewater were also confirmed to be ESBL-producers, which is in agreement with previous studies reporting that most ESBL-producing *Enterobacteriaceae* show co-resistance to fluoroquinolones, trimethoprim, and co-trimoxazole and to some aminoglycosides [38,39].

The bacterial enumeration on ESBL selective media showed that suspected ESBL-EC colonies were present in almost all studied locations (Figure S3). The amount of ESBL-EC found in the nursing home wastewater was 4.5 logs lower than the other wastewaters. Other studies showed important differences on ESBL-EC colonization rates by residents of nursing homes and long term health care facilities ranging from zero to 23.7% and from zero to 75% [40,41]. The consumption of cephalosporins was negligible in the nursing-home investigated in this study.

From the *E. coli* isolates obtained from non-selective media, only a few strains were confirmed to have the ESBL-EC phenotype (results not shown). The differences in the detection of ESBL-EC phenotypes with/without selective media illustrated the difficulty to detect rare resistance phenotypes without using selective media. 

### 3.4. Hospital Wastewater as Source for Antimicrobial Resistance in the WWTP

**Figure 3:**
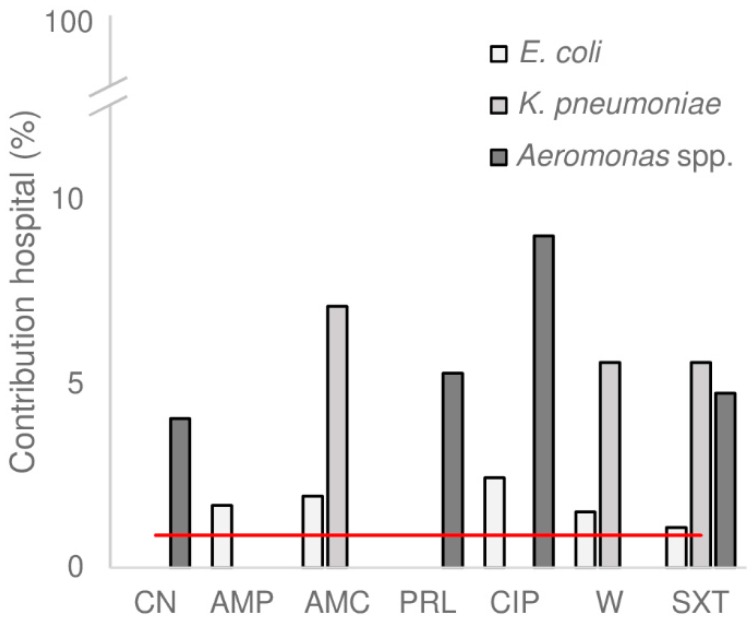
Contribution of hospital wastewater to the total wastewater. The contribution of hospital wastewater to the total volume of water treated by the wastewater treatment plant (WWTP) was approximately 1% (red line). The percentage of antimicrobial resistant bacteria in influent originating from hospital wastewater (grey bar plots) were calculated as explained in Section 2.5. CN = gentamicin, AMP = ampicillin, AMC = co-amoxiclav, PRL = piperacillin, CIP = ciprofloxacin, W = trimethoprim, SXT = co-trimoxazole. Antimicrobials with only one resistant isolate in influent were not included in the calculation.

Although hospital wastewater contained a clearly elevated concentration of resistant bacteria, the contribution of hospital wastewater to the total number of resistant bacteria in the influent was limited to a maximum of 9% (Figure 3). This is because hospital wastewater only represented a small proportion of the total influent (~1%). Similarly, Buelow et al. described that no difference in the relative abundance of antimicrobial resistance genes was found in influent from WWTPs with and without hospital sewage [5]. Therefore, pre-treatment of hospital wastewater before discharge into the sewage system might only have had a minor impact on the reduction of discharge of AMR that was being released into the environment from the sewage system.

### 3.5. All Studied Species Survived WWTP Treatment and Impacted the Receiving River

**Figure 4:**
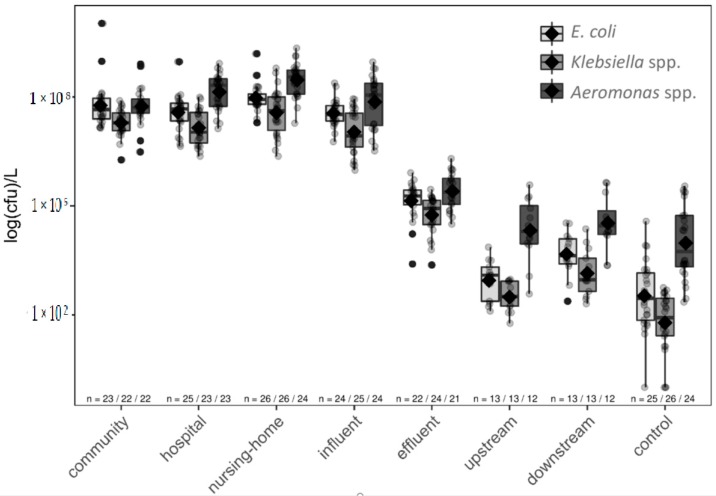
The counts of colony forming units (CFU) for the three targeted bacteria per location. The bacterial counts are shown as log (CFU)/L, *n* is the number of collected samples used for the CFU counts. The diamonds within the boxplots represent the mean, the distribution of the CFU counts are shown as scatterplots.

*E. coli, Klebsiella* spp., and *Aeromonas* spp. showed high concentrations in raw wastewater, ranging from 7 to 8 logs CFU/L (Figure 4). 

The high concentrations of *E. coli* in the raw wastewaters, which were comparable with the normal range of *E. coli* concentrations observed in other studies [42,43,44], agree with *E. coli* constituting a typical gut inhabitant [18]. Although *Aeromonas* spp. is a normal inhabitant of the intestinal tract of certain poikilotherms and freshwater fish, they are not considered to be part of the healthy human flora [36] and are found in stool of a low percentage of the population (2.5–2.6%) [37]. Thus, the high concentrations of *Aeromonas* spp. were less expected, although still these results were in agreement with a recent study [45]. A possible explanation for the high concentrations in wastewater could be that *Aeromonas* spp. persist better in the wastewater as they are aquatic bacteria. *Aeromonas* spp. has been associated with human diseases, inter alia gastroenteritis [21]. Interestingly, *Klebsiella* spp. and *Aeromonas* spp. were found in significantly higher concentrations in nursing-home wastewater compared to community, hospital, and influent (*p* < 0.05). High concentrations of these genera are only found in particular human populations suffering from diseases associated with a gut microbiome dysbiosis [34,35,36,37], which is also related to aging [46]. 

The concentration of all three bacterial species was significantly reduced after WWTP treatment (>99%) but all species were still present in effluent (~5 log CFU/L). Interestingly, an increase of *E. coli* and *Klebsiella* spp. concentrations were observed in the surface water downstream of the WWTP effluent outlet, which was significant for *E. coli* (*p* < 0.05). No significant differences in *Aeromonas* spp. concentrations upstream, downstream, and in control surface waters were observed (Figure 4), which is in line with *Aeromonas* spp. being present in the aquatic environment [47].

### 3.6. Changes in AMR in WWTP, and Influence of WWTP on Surface Water 

**Figure 5:**
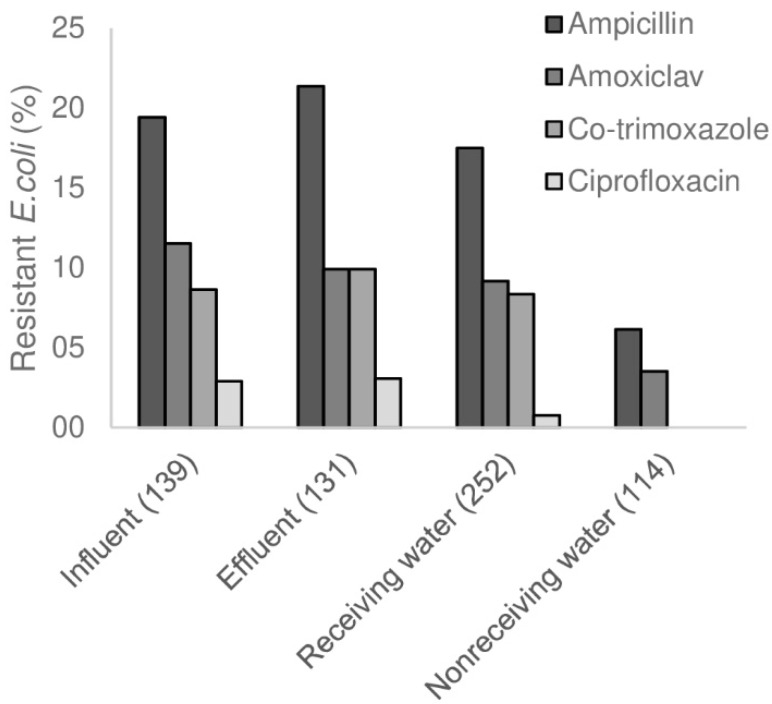
Antimicrobial resistance in the WWTP and surface water. The percentage of resistant *E. coli* against four antimicrobials in the WWTP (influent and effluent), receiving surface water and the control surface water are shown. The number of isolates tested per location are shown in brackets.

Although the concentration of all studied bacteria decreased in the WWTP by around two logs (Figure 4 and Figure S3), the percentage of resistant isolates in influent and effluent was similar (Figure 5, Table S4). Some studies indicated WWTP’s are hotspots for AMR, as the conditions in the WWTP are favourable for the propagation of AMRB [48]. A recent study showed that ARGs are decreased after wastewater treatment, but the effluents from WWTPs in southern European countries contain more ARGs than the effluents from WWTPs in northern European countries; in addition to different antibiotic consumption, temperature differences can be a possible explanation for this phenomenon [16]. In this study the percentage of resistant bacteria did neither increase nor decrease in the WWTP, indicating that there was no selection pressure for AMR in this WWTP for the bacterial species tested in this study. This finding was in concordance with another study which was also performed in a north-European country [17]. 

Antimicrobial resistance was higher in the receiving surface water compared to the control surface water (Figure 4 and Figure S3, Table S4). *E. coli* resistance against ampicillin and trimethoprim, and *Aeromonas* spp. resistance against piperacillin was significantly higher in receiving surface water versus the non-receiving surface water (*p* < 0.05). *E. coli* and *Aeromonas* spp. isolates resistant against ciprofloxacin and co-trimoxazole and ESBL-EC were only found in the receiving surface water. Only a few *Klebsiella* spp. were found in the non-receiving surface water, from which none showed resistance to any of the antimicrobials other than ampicillin. 

In the control surface water, no human markers were detected above the LOD, demonstrating that this water was in general less affected by anthropogenic activities. These results confirm that the presence of AMR, including ESBL-EC, in the surface water sites was linked to human activity. 

No significant difference between the percentage of resistant species up- and downstream waters was observed, however, bacterial enumeration showed that the WWTP had a significant effect on the concentrations of *E. coli* in surface water (*p* > 0.05). Thus, resistant bacteria might also have been present upstream the effluent site albeit in lower concentrations. Generally, this surface water was also impacted by human wastewater as it is evident from the presence of human markers upstream of the effluent discharge. Only a few isolates were retrieved from surface water which made it difficult to analyse resistance percentages in water up- and downstream from the effluent point.

Almost all antimicrobial concentrations decreased by 50% or more in the WWTP (Tables S2 and S3). The human markers, heavy metals, and six of the antimicrobial residues could still be measured in the effluent (Tables S2 and S3), and ciprofloxacin was even measured above the predicted no-effect concentrations for resistance selection (PNEC) [49]. Although none of the antimicrobials were measured above the LOD in the surface waters, human markers were found in the receiving surface water, and the concentration of gabapentin was higher downstream from the effluent point (*p* > 0.05, *t*-test). These results showed that the WWTP was continuously discharging contaminants as well as resistant bacteria into the surface water, albeit in low concentrations. Environmental contamination with antimicrobials and other pollutants can contribute to the maintenance and spread of antimicrobial resistance genes by enhancing selection pressure, depending on the concentration and bioavailability [50]. 

## 4. Conclusions

This study investigated the abundance of AMRB (*E. coli, Klebsiella* spp., and *Aeromonas* spp.), antimicrobial use, and antimicrobial concentration in clinical and non-clinical wastewater sources, the WWTP, and surface water. The highest percentage of resistant bacteria was found in the hospital wastewater, mirroring the hospital antimicrobial consumption and the measured concentrations of antimicrobials and other chemical compounds. This was also in concordance with AMR observed in clinical isolates. However, the contribution of the hospital wastewater to the antimicrobial resistance in influent was overall smaller than 10%, in accordance with the hospital wastewater comprising only 1% of the total wastewater entering the WWTP. While *E. coli* is often used as a model organism to study AMR and faecal contamination in (waste)water, *Klebsiella* spp. and *Aeromonas* spp. showed larger differences in resistance percentages between community and hospital wastewater and might therefore be better tracers for clinical inputs. Overall, concentrations of the three bacterial species decreased during the wastewater treatment, however all three species survived the treatment and the proportion of antimicrobial resistant bacteria did not decrease after the WWTP. Moreover, resistant bacteria and human markers were found in effluent and receiving surface water, demonstrating the role of the WWTP in the environmental surface water contamination.

## Supplementary Materials

Supplementary materials can be found at https://www.mdpi.com/2076-2607/7/9/312/s1.
