# Supplementary material for: Abundance and Antimicrobial Resistance of Three Bacterial Species along a Complete Wastewater Pathway
Source: Microorganisms. 2019 Sep 3;7(9):312. doi: 10.3390/microorganisms7090312 (PMC6780886; doi:10.3390/microorganisms7090312)
Supplement: Supplementary file 1 [file microorganisms-07-00312-s001.pdf]

Supplementary Materials

Table S1. Number of samples and bacterial isolates obtained per location.

Location	No. samples obtained	No. isolates obtained			
		E. coli	*Klebsiella* spp.		*Aeromonas* spp.
			K. pneumoniae	*K. oxytoca*	
Community	25	125	27	97	123
Hospital	27	134	87	46	134
Nursing-home	26	130	73	47	126
Influent	28	139	83	55	137
Effluent	27	130	96	28	134
Receiving surface water	52	252	170	46	254
Control surface water	26	87	3	5	118
Total	221	997	539	324	1,026

Table S2. LC-MS measurements of antimicrobial residues and their corresponding PNEC value (µg/L).

Sample	TRIM	SMO	CIP	CM	AZI	CLAR	ERY	DOX	TET
**PNEC (FASS)**	**56.00**	**0.59**	**1.20**	**NA**	**NA**	**0.04**	**0.10**	**NA**	**NA**
**PNEC [49]**	**0.50**	**16.00**	**0.06**	**1.00**	**0.25**	**0.25**	**1.00**	**2.00**	**1.00**
Hospital									
Samples	26	26	26	26	26	26	26	14	14
Recovery 60-140%	26	23	21	26	11	25	26	14	14
Above LOD	21	16	21	22	10	17	8	13	6
Mean (µg/L)	0.74	1.50	23.41	1.11	3.14	0.27	0.36	1.05	0.24
Consumption	10.52	6.79	97.14	1.23	0.20	0.69	0.72	1.35	0
Nursing home									
Samples	26	26	26	26	26	26	26	14	14
Recovery 60-140%	26	11	16	26	8	24	26	10	14
Above LOD	21	2	15	7	0	3	0	10	2
Mean (µg/L)	9.02	2.06	5.89	0.14	0.04	0.01	0.07	0.96	0.06
Consumption	4.39	0.83	7.62	0.54	0	0	0	5.87	0
Community									
Samples	24	24	24	24	24	24	24	14	14
Recovery 60-140%	24	21	17	24	8	23	23	14	14
Above LOD	14	2	5	4	2	0	0	13	2
Mean (µg/L)	0.45	0.23	0.57	0.03	0.24	0.01	0.07	1.13	6,96
Influent									
Samples	26	26	26	26	26	26	26	14	14
Recovery 60-140%	25	23	23	25	11	23	26	14	14
Above LOD	23	18	23	14	9	23	0	12	7
Mean (µg/L)	0.14	0.31	0.81	0.02	0.21	0.15	0.07	0.35	0.96
Effluent									
Samples	26	26	26	26	26	26	26	14	14

Recovery 60-140%	25	24	23	25	22	25	26	14	14
Above LOD	18	17	11	18	10	21	0	0	1
Mean (µg/L)	0.07	0.13	0.07	0.03	0.07	0.04	0.07	0.14	0.05

Measured antimicrobial values above one of the PNEC values are colored in red. Recovery 60-140%: the number of good measurements (recovery rates within the range of 60-140%). Above LOD: the number of the good measurements that were above LOD, which should be minimal 5 measurements. Mean (ug/L): the average of the good measurements; the value of LOD/√2 was used for measurements below LOD. TRIM = trimethoprim, SMO = sulfamethoxazole, CIP = ciprofloxacin, CM = Clindamycin, AZI = azithromycin, CLAR = clarithromycin, ERY = erythromycin, DOX = doxycycline, TET = tetracycline. Antimicrobial consumption is presented in DDD/1000-patients (hospital) or DDD/1000-residents (nursing-home).

Table S3. LC-MS and ICP measurements of human marker, heavy metals and BAC12.

Sample	ACSUL (µg/L)	GAPE (µg/L)	SUCRAL (µg/L)	Copper (µg/L)	Zinc (µg/L)	BAC12 (µg/L)
Hospital (25)						
Recovery 60-140%	21	25	6	25	25	22
Above LOD	21	24	6	23	35	22
Mean (µg/L)	15.9	4.9	15.3	102.2	34.8	18.1
Nursing home (26)						
Recovery 60-140%	10	26	4	26	26	22
Above LOD	10	0	4	26	25	11
Mean (µg/L)	78.4	0	11.8	87.4	56.2	1.5
Community (23)						
Recovery 60-140%	8	23	3	23	23	19
Above LOD	8	0	3	23	23	19
Mean (µg/L)	50.4	0	39.9	60.8	48.2	3.4
Influent (25)						
Recovery 60-140%	14	23	7	25	25	16
Above LOD	14	23	7	9	11	16
Mean (µg/L)	20.7	2.6	8.3	19.6	28.0	1.7
Effluent (23)						
Recovery 60-140%	18	23	11	NA	23	NA
Above LOD	17	23	11	NA	16	NA
Mean (µg/L)	2.4	1.5	8.8	NA	27.8	NA
Upstream (13)						
Recovery 60-140%	10	13	7	NA	NA	NA
Above LOD	10	12	6	NA	NA	NA
Mean (µg/L)	0.5	0.1	1.3	NA	NA	NA
Downstream (13)						
Recovery 60-140%	10	13	7	NA	NA	NA
Above LOD	10	13	6	NA	NA	NA
Mean (µg/L)	0.5	0.2	1.2	NA	NA	NA

The mean concentration of measured residues is calculated using the samples with the appropriate recovery percentages (60-140%). The number of samples that had measurements above LOD are shown as well, the value of LOD/√2 was used for measurements below LOD. ACSUL = acesulfame, GAPE = gabapentin, SUCRAL = sucralose, BAC12 = benzalkonium chloride 12. Grab samples (community, hospital, nursing-home, influent and effluent) were excluded for calculations.

Table S4. Antimicrobial resistance results per bacterial species and per location.

		MDR	Aminoglycosides		B-lactams			Cephalosporins			Fluoroquinolones	Sulfonamides	
**Species/genera**	**Location**		**CN**	**TOB**	**AMP**	**AMC**	**PRL**	**CXM**	**CAZ**	**CTX**	**CIP**	**W**	**SXT**
E. coli	Community (n = 125)	<0.8	0.8	0.8	10.4	6.4	-	<0.8	<0.8	<0.8	1.6	7.2	4.8
	Hospital (n = 134)	3	1.5	3.0	20.9	14.2	-	2.2	2.2	2.2	4.5	9.7	6.0
	Nursing home (n =130)	2.3	<0.8	<0.8	50.8	33.1	-	0.8	<0.8	<0.8	9.2	6.9	6.9
	Influent (n = 139)	2.9	<0.7	<0.7	19.4	11.5	-	0.7	0.7	0.7	2.9	10.1	8.6
	Effluent (n = 130)	1.5	0.8	<0.8	20.8	9.2	-	1.5	0.8	0.8	3.1	10.8	10.0
	Receiving surface water (n = 252)	<0.4	<0.4	<0.4	17.5	9.1	-	0.4	<0.4	<0.4	0.8	9.1	8.3
	Control surface water (n = 87)	<1.1	<1.1	<1.1	6.9	3.4	-	1.1	<1.1	<1.1	<1.1	<1.1	<1.1
K. pneumoniae	Community (n = 27)	<3.7	<3.7	<3.7	63.0	3.7	-	<3.7	<3.7	<3.7	<3.7	<3.7	<3.7
	Hospital (n = 87)	10.3	11.5	10.3	90.8	16.1	-	10.3	10.3	10.3	10.3	12.6	12.6
	Nursing home (n =73)	<1.4	<1.4	<1.4	95.9	4.1	-	<1.4	<1.4	<1.4	<1.4	2.7	2.7
	Influent (n = 83)	1.2	1.2	1.2	88.0	6.0	-	1.2	1.2	1.2	1.2	6.0	6.0
	Effluent (n = 96)	<1.0	1.0	1.0	81.3	4.2	-	<1.0	<1.0	<1.0	<1.0	2.1	2.1
	Receiving surface water (n = 170)	<0.6	<0.6	<0.6	84.1	3.5	-	<0.6	<0.6	<0.6	<0.6	1.2	<0.6
	Control surface water (n = 3)	<33.3	<33.3	<33.3	33.3	<33.3	-	<33.3	<33.3	<33.3	<33.3	<33.3	<33.3
K. oxytoca	Community (n = 97)	1	<1.0	<1.0	79.4	2.1	-	1.0	<1.0	<1.0	<1.0	3.1	3.1
	Hospital (n = 46)	8.7	17.4	8.7	89.1	10.9	-	8.7	8.7	8.7	8.7	2.2	2.2
	Nursing home (n =47)	<2.1	<2.1	<2.1	100.0	4.3	-	2.1	<2.1	<2.1	<2.1	2.1	<2.1
	Influent (n = 55)	<1.8	<1.8	<1.8	83.6	1.8	-	1.8	<1.8	<1.8	<1.8	<1.8	<1.8
	Effluent (n = 28)	<3.6	<3.6	<3.6	85.7	<3.6	-	<3.6	<3.6	<3.6	<3.6	<3.6	<3.6
	Receiving surface water (n = 46)	<2.2	<2.2	<2.2	91.3	4.3	-	<2.2	<2.2	<2.2	<2.2	<2.2	<2.2
	Control surface water (n = 5)	<20	<20	<20	80.0	<20	-	<20	<20	<20	<20	<20	<20

Aeromonas spp.	Community (n = 123)	<0.8	1.6	-	-	-	2.4	-	0.8	-	<0.8	-	2.4
	Hospital (n = 134)	15.7	13.4	-	-	-	30.6	-	14.9	-	22.4	-	31.3
	Nursing home (n =126)	0.8	1.6	-	-	-	2.4	-	<0.8	-	15.9	-	21.4
	Influent (n = 137)	1.5	2.9	-	-	-	5.1	-	0.7	-	2.2	-	5.8
	Effluent (n = 134)	<0.7	0.7	-	-	-	9.0	-	<0.7	-	0.7	-	6.0
	Receiving surface water (n = 254)	0.8	0.4	-	-	-	7.1	-	2.0	-	1.6	-	4.3
	Control surface water (n = 118)	<0.8	4.2	-	-	-	0.8	-	0.8	-	<0.8	-	<0.8

Percentages of antimicrobial resistance found per bacterial species and per location. MDR = multi-drug resistance, CN = gentamicin, TOB = tobramycin, AMP = ampicillin, AMC = co-amoxiclav, PRL = piperacillin, CXM = cefuroxime, CAZ = ceftazidime, CTX = cefotaxime, CIP = ciprofloxacin, W = trimethoprim, SXT = co-trimoxazole.

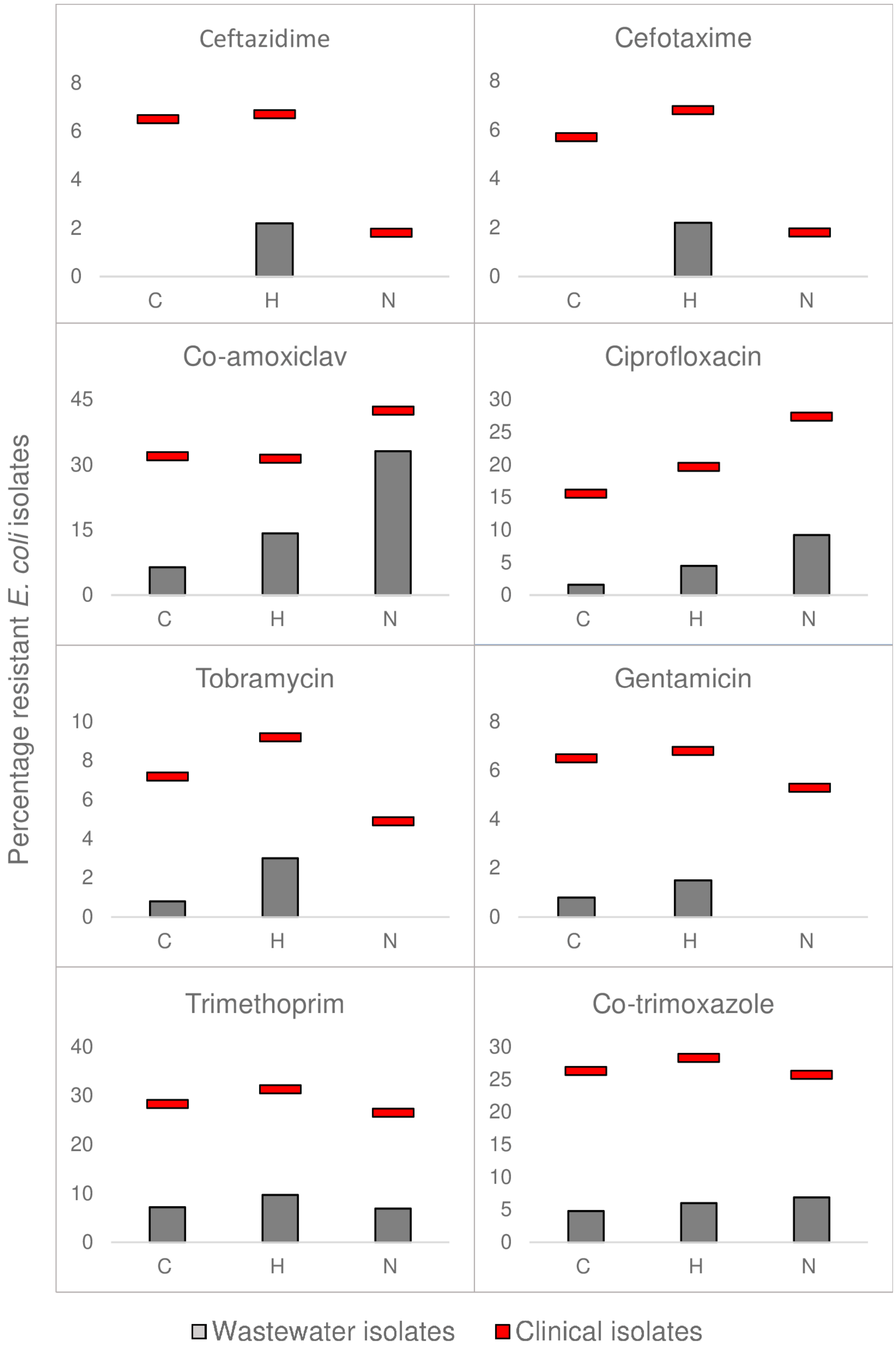

Figure S1. Antimicrobial resistance percentages of clinical *E. coli* isolates vs wastewater *E. coli* isolates. C = community, H = hospital, N = nursing-home.

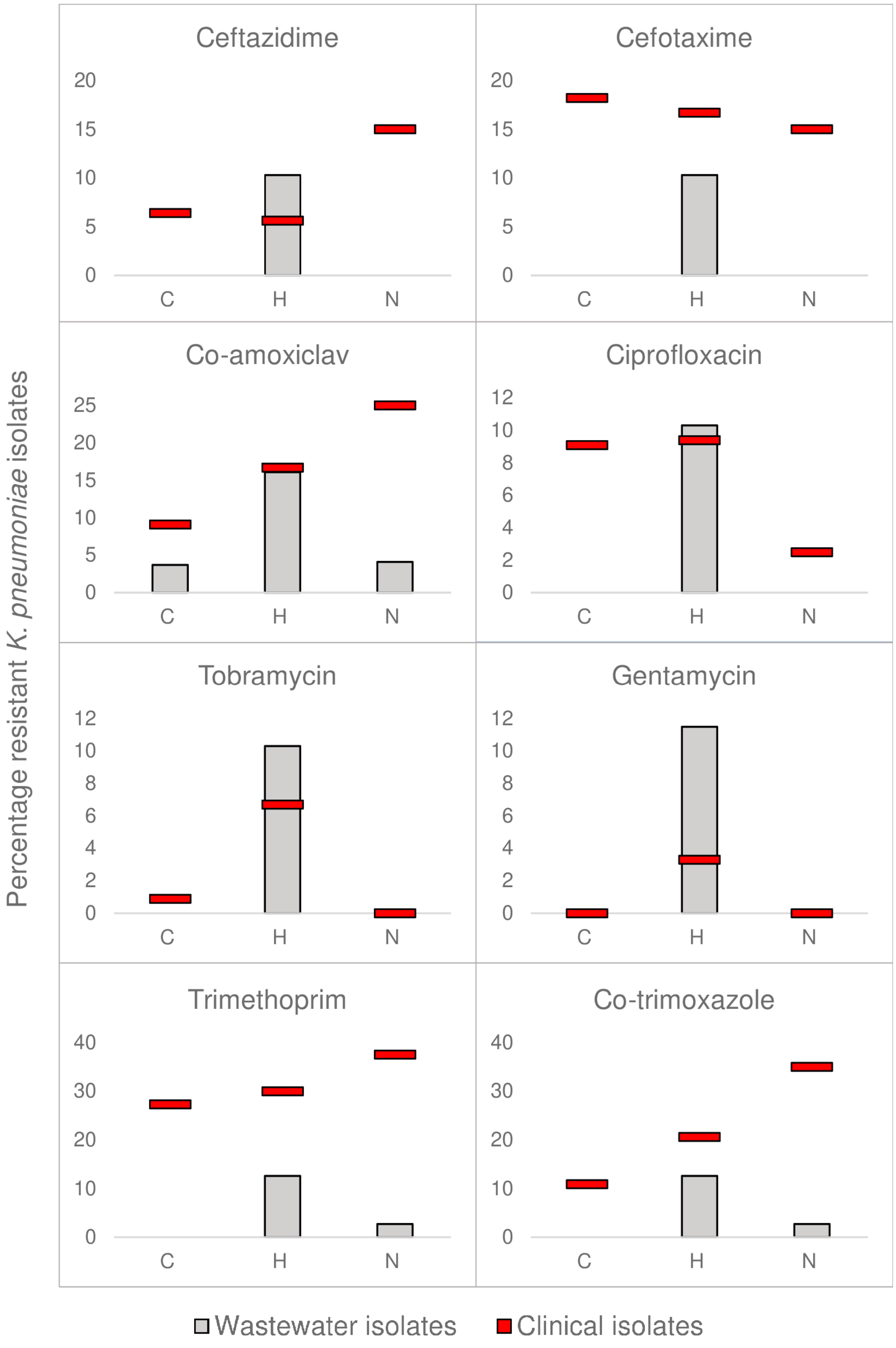

Figure S2. Antimicrobial resistance percentages of clinical *K. pneumoniae* isolates vs wastewater *K. pneumoniae* isolates. C = community, H = hospital, N = nursing-home.

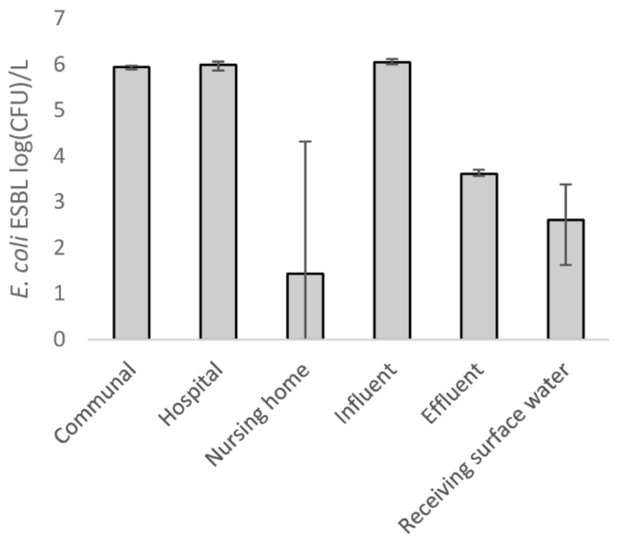

Figure S3. ESBL-producing *E. coli* counts. CFU-counts of ESBL-producing E. coli in the different water samples collected during the first sampling-days of September, October and November 2017. No ESBL-EC were observed in the control surface water.
